# Probiotic *Lactobacillus casei* improves immune microenvironment in rheumatoid arthritis via gut microbiota-butyrate-HDAC/NF-κB signaling

**DOI:** 10.1080/19490976.2026.2698969

**Published:** 2026-07-21

**Authors:** Yanmiao Ma, Mingyang Li, Liyuan Jian, Jiehao Peng, Ya Wen, Yuanhui Hao, Jiajia Shu, Yali Song, Baozhen Li, Chengwu Zhang, Xianyu Li, Guanglin Li, Yonghui Wang, Tao Peng, Ran Zhou

**Affiliations:** a College of Basic Medical Sciences, Shanxi University of Chinese Medicine, Taiyuan, People's Republic of China; b Shanxi Provincial Key Laboratory of Classical Prescription Strengthening Yang, Taiyuan, People's Republic of China; c Shanxi Provincial Key Laboratory of Prescription Compatibility and Functions, Taiyuan, People's Republic of China; d College of First Clinical Medicine, Shanxi University of Chinese Medicine, Taiyuan, People's Republic of China; e College of Third Clinical Medicine, Shanxi University of Chinese Medicine, Taiyuan, People's Republic of China; f Heping Hospital affiliated to Changzhi Medical College, Changzhi, People's Republic of China; g School of Basic Medical Sciences, Shanxi Medical University, Taiyuan, People's Republic of China; h Beijing Key Laboratory of Traditional Chinese Medicine Basic Research on Prevention and Treatment for Major Diseases, Experimental Research Center, China Academy of Chinese Medical Sciences, Beijing, People's Republic of China; i Key Laboratory of Ministry of Education for Medicinal Plant Resource and Natural Pharmaceutical Chemistry, National Engineering Laboratory for Resource Development of Endangered Crude Drugs inNorthwest China, College of Life Sciences, Shaanxi Normal University, Xi'an, People's Republic of China; j Shanxi Hospital of Integrated Traditional Chinese and Western Medicine, Taiyuan, People's Republic of China

**Keywords:** Probiotic intervention, *Lactobacillus casei*, short-chain fatty acids, gut–joint axis, anti-inflammatory activity

## Abstract

Rheumatoid arthritis (RA) is a chronic autoimmune disorder characterized by persistent synovial inflammation, progressive joint destruction, and functional disability. Emerging evidence indicates that gut microbiota dysbiosis and host protein glycosylation play essential roles in regulating immune signaling during RA progression. In this study, we demonstrate that the probiotic strain *Lactobacillus casei* (abbreviated as *L. casei*) modulates gut microbial composition and enhances butyrate production, consequently impacting HDAC/NF-κB signaling and O-GlcNAc glycosylation. The administration of *L. casei* significantly alleviated arthritis symptoms and synovial damage, reduced serum inflammatory cytokine levels, restructured the gut microbiota structure, and enhanced butyrate production. Moreover, *L. casei* markedly increased O-GlcNAcylation of key immune signaling proteins, such as STAB1, by downregulating O-GlcNAcase (OGA) activity. Importantly, *L. casei* also modulated histone deacetylase (HDAC) expression and inhibited NF-κB pathway activation, synergistically contributing to glycosylation-mediated anti-inflammatory effects. This study provides evidence that *L. casei* alleviates synovial inflammation and contributes to immune homeostasis in rheumatoid arthritis by collectively improving the joint immune microenvironment and mitigating inflammatory responses.

## Graphical abstract

**Figure:**
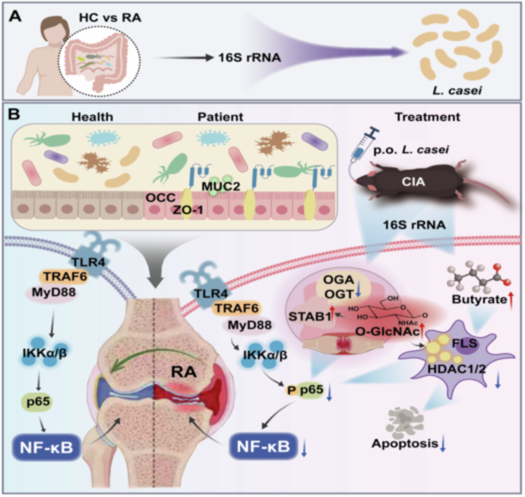

## Introduction

As a systemic autoimmune disorder, Rheumatoid arthritis (RA) is characterized by synovial hyperplasia and bone erosion, resulting in joint damage and deformity. This debilitating condition imposes significant health and economic burdens, evidenced by a projected global RA treatment market of $33.96 billion by 2025.^1^ The pathology of RA involves abnormal immune activation, driven by key players such as pro-inflammatory cytokines (TNF-*α*, IL-1β, IL-6) and immune cells, leading to synovial inflammation and bone destruction.^2^ Histone deacetylases (HDACs) are crucial for epigenetic regulation, influencing gene transcription and signaling by deacetylating both histone and non-histone proteins.^3^ High expression levels of HDAC1/2 in the RA synovium correlate with increased inflammatory factors, FLS proliferation, and joint destruction.^4^ HDACs amplify inflammatory signaling via the NF-κB, STAT3, and MAPK pathways, consequently perpetuating chronic inflammation.^5^ HDAC inhibitors can alleviate inflammation and inhibit FLS invasion, positioning them as a promising epigenetic-targeted therapeutic strategy for RA.^6^

Gut microbiota dysbiosis is also pivotal in the pathogenesis of RA, characterized by a decrease in beneficial bacteria *(e.g., Faecalibacterium prausnitzii, Bifidobacterium,* and *Lactobacillus*) and an increase in potential pathogens *(e.g., Prevotella copri,* and *Eggerthella lenta*). This imbalance disrupts the intestinal barrier, increasing its permeability, and thereby promoting the translocation of LPS and citrullinated proteins, which can trigger systemic inflammation. Beyond this, microbial metabolites—including SCFAs, bile acids, and indole derivatives—are crucial for regulating T cell differentiation, macrophage polarization, and NF-κB signaling.^7^ Modulating gut microbiota and its metabolites may offer a potential strategy for alleviating RA. For instance, *Lactobacillus casei* has demonstrated anti-inflammatory effects, regulates T cell balance, and enhances mucosal barriers.^8^ By reshaping gut microbiota and restoring immune homeostasis, *L. casei* may influence the gut-joint axis, where microbial metabolites serve as mediators connecting gut and joint immune responses.^9^ These metabolites can also modulate immune cell functions, inflammatory pathways, and host metabolism.^10^

O-GlcNAcylation, a reversible post-translational modification (PTM), critically regulates immune homeostasis, inflammation, and metabolic signaling. Unlike traditional glycans, O-GlcNAc involves the rapid and reversible addition of a single *N*-acetylglucosamine to nuclear or cytoplasmic proteins, thereby influencing their stability, localization, interactions, and transcription.^11^ In RA, the interplay between O-GlcNAcylation and phosphorylation, particularly on NF-κB p65 (RelA), enhances nuclear translocation and DNA binding, leading to sustained expression of TNF-*α*, IL-1β, and IL-6, which promotes synovial proliferation and inflammation.^12^ ^,^ ^13^ The O-GlcNAc modification of p65 contributes to aberrant NF-κB activation in chronic inflammation. *Lactobacillus casei* may indirectly modulate O-GlcNAcylation by reshaping the gut microbiota. This reshaping leads to increased SCFAs such as butyrate, and enhanced hexosamine biosynthetic pathway (HBP) activity, which collectively boost UDP-GlcNAc supply. Beyond this, SCFAs also regulate O-GlcNAcase (OGA) via AMPK and acetyl-CoA, thereby maintaining a dynamic O-GlcNAc balance and linking metabolism to immunity.^14^ Dysregulation of this PTM may drive RA progression.

*Lactobacillus casei* enhances host immune and metabolic status by reshaping the gut microbiota and increasing SCFAs, particularly butyrate. Its anti-inflammatory properties stem from both metabolic regulation and its ability to modulate host protein glycosylation, which impacts key signaling pathways. The HDAC/NF-κB pathway, a central regulator of inflammation, and immune imbalance, is a crucial target. Butyrate, a natural HDAC inhibitor, acts synergistically with *L. casei* to epigenetically suppress the expression of inflammatory factors. Thus, *L. casei* may regulate RA inflammation through a multi-faceted network involving the gut microbiota, glycosylation, the HDAC/NF-κB pathway, and butyrate. However, systematic studies exploring this comprehensive mechanism are limited. This study aims to investigate *L. casei' s effects* on RA via the gut-joint axis, specifically focusing on its regulation of host glycosylation and HDAC/NF-κB signaling, thereby laying the groundwork for probiotic-based interventions in RA (Figure 8F).

## Results

### Analysis of gut microbiota differences and baseline data collection between patients and healthy subjects

**Figure 1.**
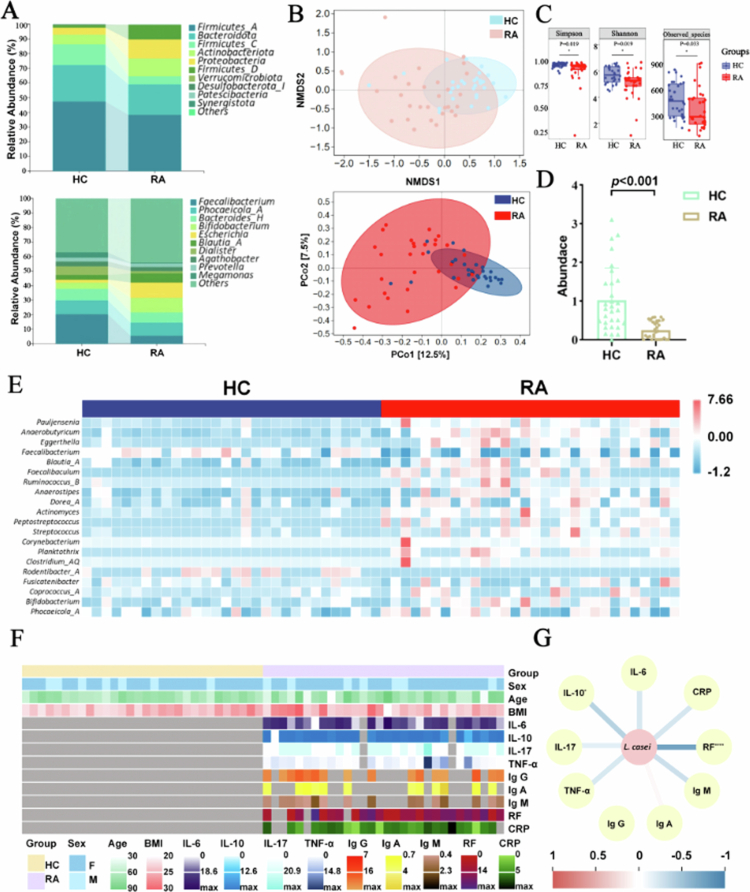
Gut microbiota alterations and clinical associations in patients with rheumatoid arthritis (RA) and healthy controls (HC). (A) Relative taxonomic composition at the phylum and genus levels in HC and RA groups (*n* = 30 per group); (B) Alpha diversity analysis, including Observed-species, Shannon, and Simpson indices; (C) Beta diversity analysis based on principal coordinates analysis (PCoA) of HC and RA groups; (D) Relative abundance of *L. casei* in HC and RA groups; (E) Random forest analysis identifying discriminative genus-level microbial taxa between groups; (F, G) Correlation analyzes between *L. casei* abundance and clinical, serological, or inflammatory parameters.

By enhancing regulatory T-cell differentiation and inhibiting IL-17, *L. casei* helps maintain immune system stability. We identified significant differences in gut microbiota between 30 healthy controls (HC) and 30 patients with RA. The relative abundance of Faecalibacterium, a key anti-inflammatory genus, was markedly reduced in RA patients (Figure 1A). Alpha diversity, measured by Observed-species (*p* = 0.019), Shannon (*p* = 0.019), and Simpson indices (*p* = 0.033), differed significantly between the groups (Figure 1B). Beta diversity analyzes based on PCoA and NMDS demonstrated distinct microbial community structures in the HC and RA groups (Figure 1C, Figure S1B, Supporting Information). LEfSe analysis revealed an overall pro-inflammatory shift in RA, characterized by reduced SCFA-producing bacteria (e.g., *Lactobacillus*, *Bifidobacterium adolescentis, Butyricimonas faecalis,* and *Roseburia*) and increased pathogenic taxa, including *Enterocloster aldenensis, Hungatella,* and *Parvimonas micra* (Figure S1A, Supporting Information). Random forest analysis identified significant intergroup differences in several SCFA-producing genera (Figure 1E). Notably, *L. casei* abundance was significantly lower in RA patients (*p* < 0.001). Correlation analysis revealed a strong negative association between *L. casei* and RF, as well as inverse trends with pro-inflammatory cytokines, confirming its role in modulating immune balance (Figure 1D, F−G, Figure S2 Table 1, Supporting Information).

### Colonization with *Lactobacillus casei* significantly alleviated rheumatoid arthritis symptoms

**Figure 2.**
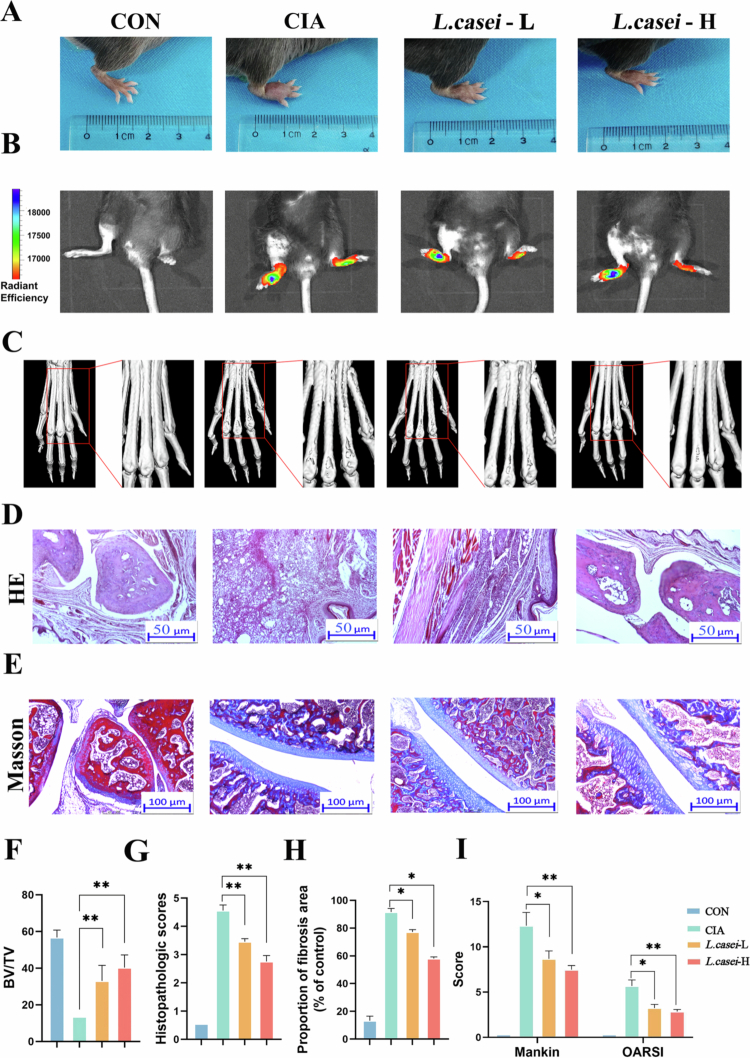
Colonization with *Lactobacillus casei* significantly alleviated arthritis symptoms in CIA mice. (A) Representative images and assessment of paw swelling after *L. casei* treatment; (B) In vivo fluorescence imaging (IVIS) of joint inflammatory signals; (C, F) Micro-CT results and quantitative analysis; (D, G) H&E staining and quantitative analysis of ankle joints; (E, H) Masson trichrome staining and statistical analysis; (I) Mankin cartilage scoring and OARSI histopathological scoring. Note: Control, normal mice; CIA, collagen-induced arthritis mice; *L. casei*-L, CIA + low-dose *L. casei*; *L. casei*-H, CIA + high-dose *L. casei.* Data are presented as mean ± SEM. One-way ANOVA followed by Tukey’ s test. (*p < 0.05; **p < 0.01).

Next, we evaluated the anti-arthritic effects of *Lactobacillus casei* using the CIA mouse model. The CIA mice exhibited severe symptoms, including paw redness, swelling, joint deformity, and stagnation or loss of body weight. After 28 days of *L. casei* treatment, joint swelling and paw thickness were notably reduced (Figure 2A, Figure S2B, C, Supporting Information). H&E staining revealed significant inflammatory infiltration, fibroblast growth, new blood vessel formation, cartilage damage, and pannus development in CIA mice. *L. casei* treatment significantly mitigated these issues (Figure 2D, G), as evidenced by improved Mankin and OARSI scores (Figure 2I). Masson staining showed increased collagen deposition and joint injury in CIA mice, while *L. casei* treatment alleviated these changes (Figure 2E, H). Micro-CT analysis demonstrated severe ankle bone erosion in the CIA mice; however, *L. casei* partially mitigated bone loss, although complete restoration was not achieved (Figure 2C, F). Finally, IVIS imaging revealed strong inflammatory fluorescence signals in the CIA joints, which were significantly reduced after *L. casei* administration, indicating decreased inflammation (Figure 2B). Collectively, these results demonstrate that *L. casei* alleviates joint inflammation and bone destruction through modulation of the gut-joint axis.

### *Lactobacillus casei* reverses gut microbiota dysbiosis

**Figure 3.**
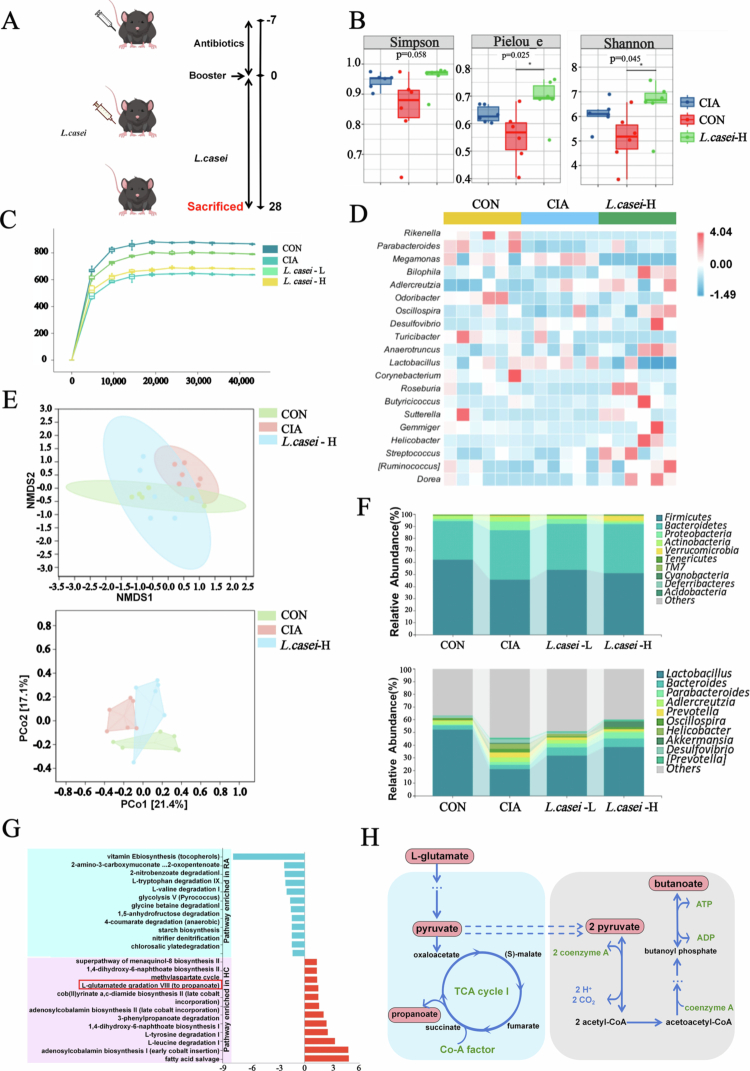
*Lactobacillus casei* reverses gut microbiota dysbiosis in CIA mice. (A) Experimental design for evaluating the effects of *L. casei* on gut microbiota in CIA mice; (B) Alpha diversity analysis, including Simpson, Pielou_e, and Shannon indices; (C) Rarefaction curves of gut microbiota across groups; (D) Random forest analysis identifying differential genus-level taxa among groups; (E) Beta diversity analysis based on PCoA and NMDS; (F) Relative bacterial abundance at the phylum and genus levels across groups; (G) PICRUSt2-predicted MetaCyc metabolic pathway analysis; (H) Reconstruction of propionate and butyrate-related microbial metabolic pathways. Note: CON, normal control mice; CIA, collagen-induced arthritis mice; *L. casei*-L, CIA + low-dose *L. casei*; *L. casei*-H, CIA + high-dose *L. casei.* (*n* = 6 mice/group).

The reversal of microbial dysbiosis by *L. casei* was investigated in the CIA mouse model (Figure 3A). 16S rRNA sequencing of fecal samples from C57BL/6J mice CON, CIA, *L. casei*-H, and *L. casei*-L groups) after 4 weeks revealed marked changes in gut microbiota. At the phylum level, the anti-inflammatory phylum Firmicutes was significantly reduced after CIA induction but markedly increased following *L. casei*-H treatment. At the genus level, beneficial bacteria such as *Lactobacillus* were decreased in the CIA group but restored after *L. casei*-H administration, along with an increase in *Akkermansia*. In contrast, harmful genera including Prevotella and Desulfovibrio were elevated in CIA mice and significantly reduced by *L. casei*-H treatment (Figure 3F). Alpha diversity, measured by Pielou_e (*P* = 0.025) and Simpson indices (*P* = 0.033), demonstrated significant differences among groups (Figure 3B, C). Beta diversity analyzes based on PCoA and NMDS demonstrated distinct microbial compositions between the CIA and *L. casei*-H groups (Figure 3E). LEfSe analysis indicated a pro-inflammatory microbial profile in CIA mice, characterized by a decrease in SCFA-producing bacteria (e.g., *Megamonas*) and an increase in pathogenic taxa. (Figure S1C, Supporting Information). Following treatment, butyrate-producing taxa, specifically *Butyricicoccus* and *Pullicaecorum*, were markedly enriched. Random forest analysis further confirmed the enrichment of SCFA-producing genera in the *L. casei*-H group (Figure 3D). PICRUSt2 and MetaCyc pathway analyzes revealed an enhancement of SCFA-related metabolic pathways. Pathway reconstruction using the BIOCYC database (PATHWAY: CENTFERM-PWY) further demonstrated that pyruvate a central intermediate can be further fermented by gut microbiota to generate butyrate, in addition to propionate production. This suggests that *L. casei* promotes the production of propionate and butyrate by modulating the structure and function of gut microbiota (Figure 3G, H).

### *Lactobacillus casei* alleviates arthritis by restoring mucosal barrier function and suppressing inflammatory responses

**Figure 4.**
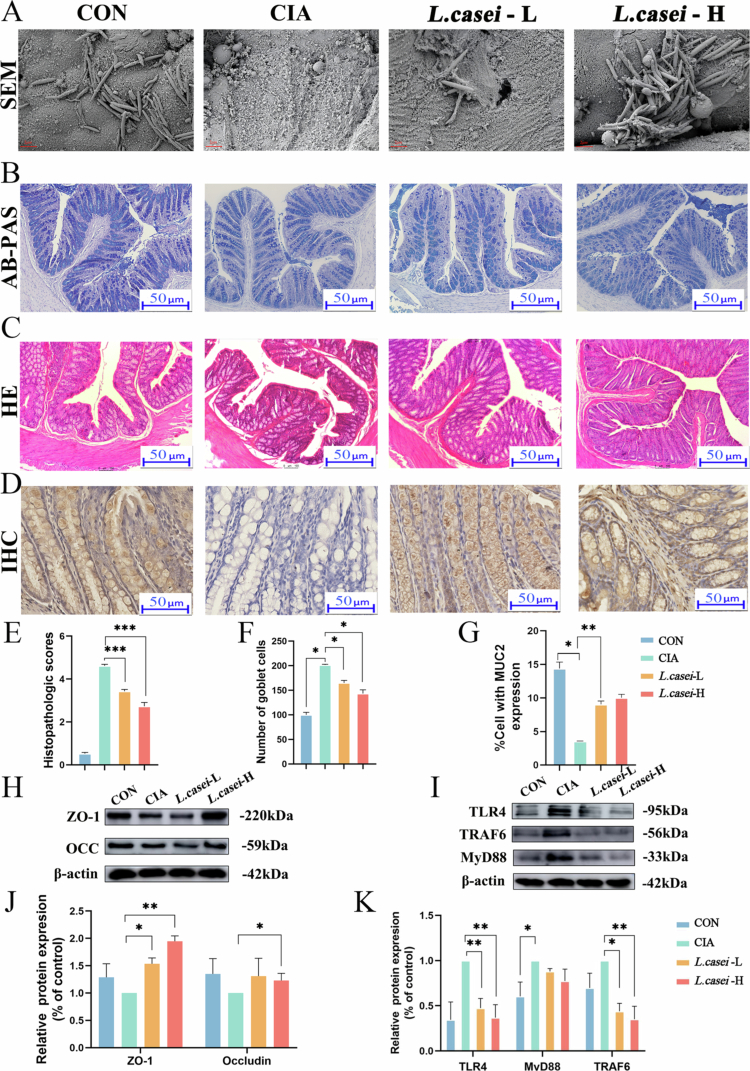
*Lactobacillus casei* alleviates arthritis by restoring intestinal barrier integrity and suppressing inflammatory responses. (A) Representative SEM images of mouse colonic mucosa; (B, F) AB-PAS staining of colonic tissue and quantitative analysis; (C, E) H&E staining of colonic tissue and histological quantification; (D, G) Immunohistochemical staining of MUC2 and quantitative analysis; (H, J) Western blot analysis of tight junction proteins (ZO-1 and Occludin) and corresponding quantification after *L. casei* intervention; (I, K) Western blot analysis of inflammatory signaling proteins (TLR4, MyD88, and TRAF6) and corresponding quantification after *L. casei* intervention. Note: CON, normal control mice; CIA, collagen-induced arthritis mice; *L. casei*-L, CIA + low-dose *L. casei*; *L. casei*-H, CIA + high-dose *L. casei*. Data are presented as mean ± SEM. (*n* = 3, *p < 0.05; **p < 0.01).

The intestinal barrier plays a crucial role in maintaining host-microbiota homeostasis. In autoimmune diseases such as RA, dysfunction of this barrier is often characterized by gut dysbiosis, reduced tight junction (TJ) proteins, and increased intestinal permeability, thereby allowing microbial products to trigger systemic inflammation. To investigate whether *L. casei* can restore barrier function during arthritis progression, colonic tissues were examined. SEM revealed that CIA mice displayed significant villus loss, epithelial damage, and pore formation. In contrast, treatment with *L. casei* significantly improved villus integrity, with both cocci and bacilli adhering to the mucosal surface, suggesting a restoration of the physical barrier (Figure 4A). Western blot analysis showed that *L. casei* markedly increased the colonic expression of TJ proteins ZO-1 and Occludin while suppressing inflammatory signaling proteins TLR4, TRAF6, and MyD88 (Figure 4H−K). AB/PAS staining indicated a pronounced decrease in goblet cells and abnormal acidic mucus secretion in CIA mice, both of which were alleviated by *L. casei* treatment, reflecting improved mucosal barrier integrity (Figure 4B, F). H&E staining of colonic tissue revealed architectural damage and inflammatory cell infiltration in CIA mice, which were ameliorated by *L. casei* (Figure 4C, E). Immunohistochemical analysis showed that MUC2 expression in the colonic epithelium was decreased in CIA mice but markedly increased after *L. casei* administration (Figure 4D, G), indicating enhancement of chemical and immune barrier functions.

### *Lactobacillus casei* inhibits HDAC/NF-κB signaling pathway proteins and modulates related inflammatory factors

**Figure 5.**
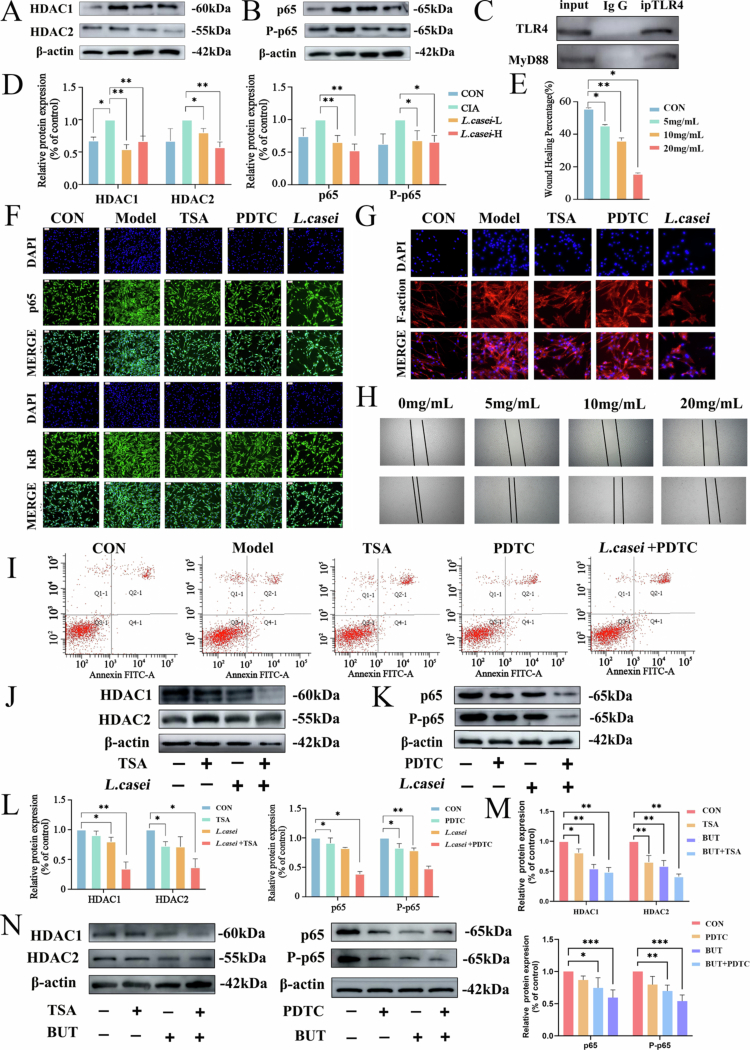
*Lactobacillus casei* inhibits HDAC/NF-κB signaling and modulates inflammatory responses. (A, B, D) Western blot analysis of HDAC1, HDAC2, p65, and phosphorylated p65 in synovial tissues after *L. casei* treatment; (C) Co-immunoprecipitation analysis of TLR4-MyD88 interaction; (E, H) Wound-healing assay of FLS migration after heat-inactivated *L. casei* treatment (0, 5, 10, and 20 mg/mL) and corresponding quantification; (F, G) Immunofluorescence and phalloidin staining of FLS cells after indicated treatments; (I) Flow cytometry analysis of apoptosis in FLS cells; (J, K, L) Western blot analysis of HDAC1, HDAC2, p65, and phosphorylated p65 in FLS cells and quantification; (M, N) Western blot analysis of HDAC1, HDAC2, p65, and phosphorylated p65 in RA-FLS cells treated with But alone or combined with TSA/PDTC, and quantification. Note: Model, RA-FLS inflammatory model; CIA, collagen-induced arthritis; TSA, Trichostatin A; PDTC, pyrrolidine dithiocarbamate; But, sodium butyrate; *L. casei*-L, CIA + low-dose *L. casei*; *L. casei*-H, CIA + high-dose *L. casei*. (*n* = 3, *p < 0.05; **p < 0.01).

To clarify the effects of *L. casei* on the NF-κB pathway and its interaction with TLR4/MyD88 signaling, western blot analysis showed that *L. casei* markedly reduced the expression of HDAC1, HDAC2, p65, and phosphorylated p65 in synovial tissues compared with the CIA group, indicating suppression of HDAC/NF-κB signaling (Figure 5A, B, D). Co-immunoprecipitation demonstrated a significant reduction in the interaction between TLR4 and MyD88 following *L. casei* treatment, indicating inhibition of downstream TLR4/MyD88 signaling (Figure 5C). Wound-healing assays further demonstrated that *L. casei* inhibited FLS migration in a dose-dependent manner (Figure 5E, H). Immunofluorescence and phalloidin staining further showed that *L. casei* attenuated inflammatory activation and cytoskeletal remodeling (Figure 5F, G). Flow cytometry analysis showed that TSA and PDTC both increased FLS apoptosis compared with the model group, while combined treatment with *L. casei* and PDTC significant decreased apoptosis relative to PDTC alone (Figure 5I). Consistently, western blot analysis confirmed reduced expression of HDAC1, HDAC2, p65, and phosphorylated p65 following *L. casei* treatment, with stronger inhibition observed after co-treatment with TSA or PDTC (Figure 5J, K, L). To further validate butyrate's causal role in this regulatory pathway, RA-FLS cells were treated with But alone or in combination with TSA and PDTC. Western blot analysis showed that But significantly reduced the expression of HDAC1, HDAC2, P65, and phosphorylated P65, while combined treatment exerted stronger inhibitory effects on HDAC/NF-κB signaling (Figure 5M, N). Collectively, these findings suggest that *L. casei* mitigates arthritis progression by targeting the HDAC/NF-κB and TLR4/MyD88 pathways, with butyrate acting as an important functional mediator.

### O-GlcNAc proteomics

**Figure 6.**
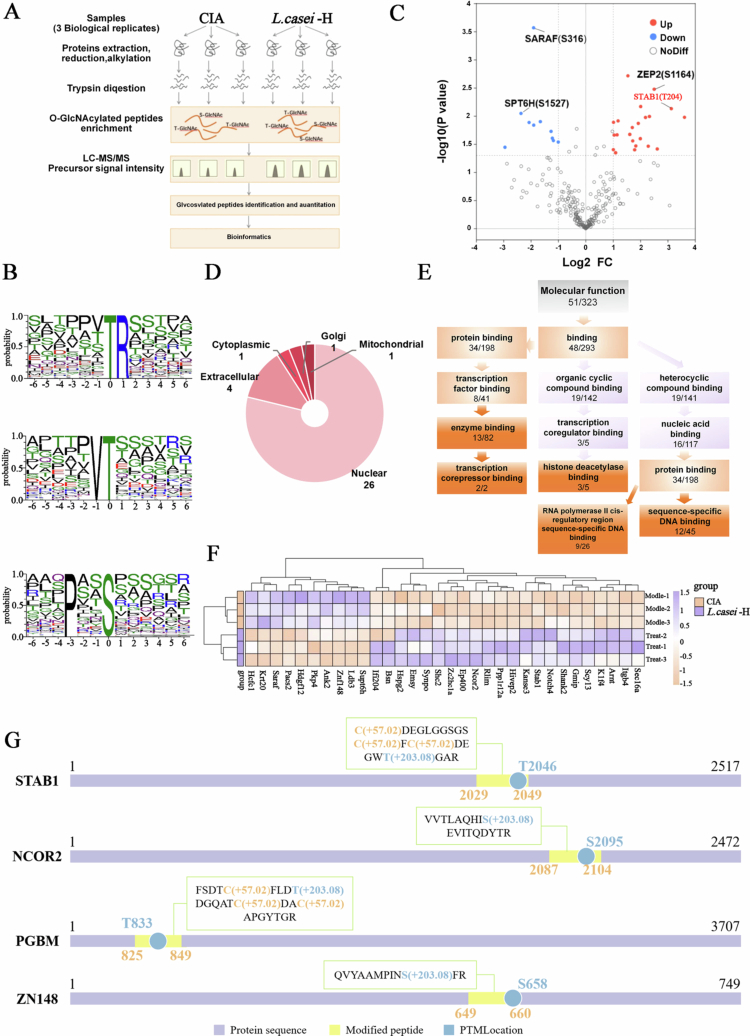
O-GlcNAc Glycosylated Proteomics. (A) Experimental workflow diagram; (B) Peptide motif identification; (C) Volcano plot (red indicates upregulation, blue indicates downregulation, gray indicates no significant change); (D) Subcellular localization prediction of differentially expressed peptides; (E) GO enrichment analysis of differentially expressed peptides (color scale: gray, purple, orange-yellow, orange indicate increasing enrichment levels); (F) Hierarchical clustering heatmap of O-GlcNAc-modified peptides; (G) Schematic diagram of peptide modification sites (purple: amino acid sequence of protein; yellow: modified peptide segment; blue: position of modification site within protein sequences). Note: CIA, collagen-induced arthritis mice; *L. casei*-H, CIA + high-dose *L. casei*.

O-GlcNAc is a reversible and dynamic post-translational modification occurring on serine or threonine residues, and it has been implicated in inflammation-related diseases. To investigate its potential role in RA, we performed O-GlcNAc proteomic analysis using colonic tissues from CIA and *L. casei*-H mice. The overall experimental workflow is depicted in Figure 6A. Motif analysis suggested that these modified serine and threonine residues may serve as important regulatory sites, particularly within cell signaling domains (Figure 6B). Through fold change and *P* value screening, we identified 32 differentially O-GlcNAc-modified peptides, comprising 22 that were upregulated and 10 that were downregulated (Figure 6C). Hierarchical clustering further demonstrated distinct O-GlcNAc modification patterns between the CIA and *L. casei*-H groups (Figure 6F). Subcellular localization analysis showed that the corresponding proteins were predominantly nuclear (Figure 6D). GO enrichment analysis indicated significant enrichment in biological processes related to cellular component maintenance and synaptic structure, and in molecular functions associated with promoter-specific DNA binding and synapse-related cellular components (Figure 6E, Figure S2A, Supporting Information). Domain annotation also suggested an enrichment of Zinc finger C2H2-type domains. Additionally, site distribution analysis identified a conserved O-GlcNAc modification at threonine 2046 of STAB1 (Figure 6G), a modification shared between humans and mice that may participate in cell adhesion and signal transduction. Other modified proteins similarly demonstrated functional relevance, further supporting the potential importance of O-GlcNAc regulation in RA pathogenesis.

### Glycosylation database analysis and related proteins

**Figure 7.**
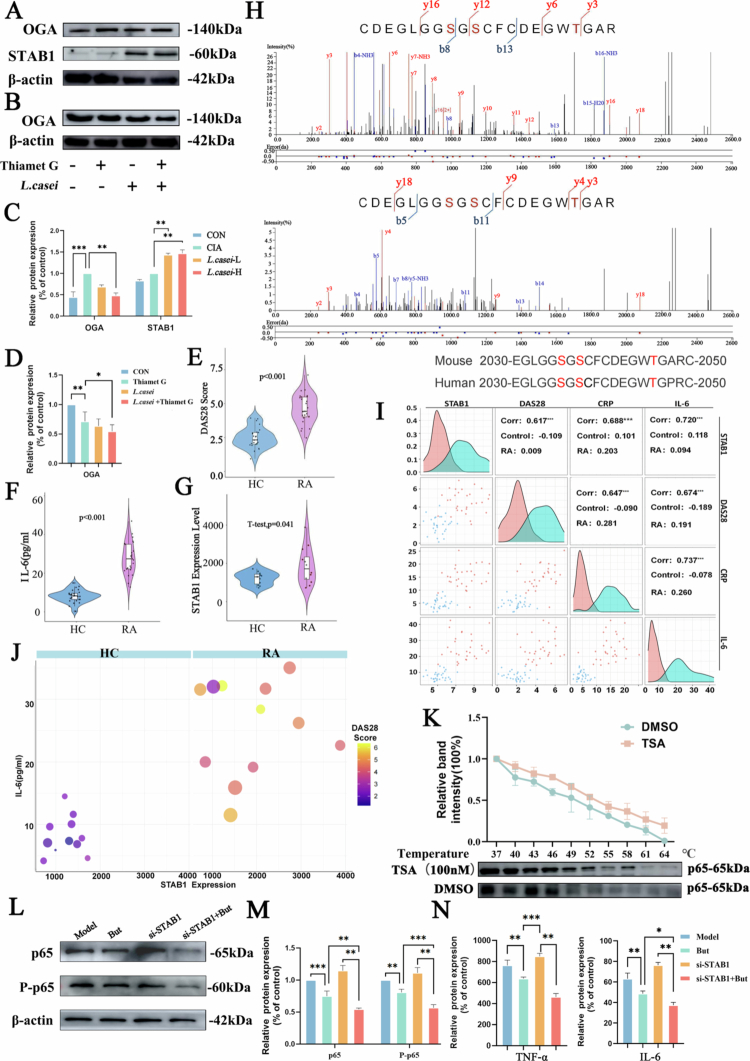
Glycosylation database analysis and related protein validation. (A, C) Western blot analysis of STAB1 and OGA expression after *L. casei* intervention; (B, D) Analysis of FLS cells after treatment with *L. casei* combined with Thiamet G; (E, F, G) GEO database analysis of STAB1, DAS28, and IL-6; (H) Secondary mass spectrometry of STAB1 modification sites; (I, J) Correlation analysis of DAS28, IL-6, and STAB1; (K) CETSA analysis of NF-κB p65 protein stability; (L, M) Western blot analysis and quantitative analysis of p65 and P-p65 expression in RA-FLS treated with But, with or without STAB1 knockdown (si-STAB1); (*N*) ELISA analysis of inflammatory cytokines after indicated treatments. Note: CIA, collagen-induced arthritis mice; *L. casei*-L, CIA + low-dose *L. casei*; *L. casei*-H, CIA + high-dose *L. casei*; But, sodium butyrate; Thiamet G, OGA inhibitor. (*n* = 3; GEO dataset: RA *n* = 16, HC *n* = 7; *p < 0.05; **p < 0.01; ***p < 0.001).

Leveraging the O-GlcNAc modification profile, we integrated clinical data and experimental validation to assess biological relevance. We downloaded synovial tissue transcriptomic data from the Gene Expression Omnibus (GEO; https://www.ncbi.nlm.nih.gov/geo/) under accession number GSE77298. This dataset originates from Broeren MG’s prospective RA cohort collected in the Netherlands in 2016, comprising synovial samples from 16 RA patients and 7 healthy controls. We extracted and analyzed baseline clinical parameters alongside STAB1 expression. Notably, RA patients with elevated STAB1 expression exhibited significantly higher DAS28 scores and IL-6 levels compared to healthy controls. Furthermore, both DAS28 and IL-6 levels were positively correlated with STAB1 expression (Figure 7E−G, I, J), suggesting STAB1 as a potential diagnostic target.

Proteomic analysis revealed significant alterations in the glycosylation of STAB1 in the RA model, suggesting its involvement in inflammation-related pathways (Figure 7H). Western blot analysis confirmed that STAB1 protein expression was increased in CIA mice compared to controls, aligning with increased glycosylation levels. Western blot analysis further showed that OGA expression were significantly increased in CIA mice compared with controls, whereas *L. casei* treatment reduced OGA expression (Figure 7A, C). In FLS cells, combined treatment with *L. casei* and the OGA inhibitor Thiamet G further enhanced O-GlcNAc modification and modulated inflammatory signaling–related protein expression (Figure 7B, D). CETSA analysis revealed reduced thermal stability of NF-κB p65 in CIA mice, suggesting conformational alterations potentially driven by abnormal O-GlcNAc modification, which may impact its transcriptional activity (Figure 7K). Collectively, these findings imply that RA-associated inflammation modulates glycosylation enzymes, thus altering protein modification and function, including that of STAB1. To further define the functional role of STAB1, siRNA-mediated knockdown was performed in RA-FLS cells. STAB1 silencing enhanced NF-κB signaling activity, while butyrate treatment exerted an inhibitory effect (Figure 7L, M). Notably, butyrate partially attenuated the effect induced by STAB1 knockdown, suggesting a functional interplay between STAB1 and butyrate in regulating this pathway. Consistent results were observed in the ELISA analysis of downstream inflammatory mediators (Figure 7N). These findings indicate STAB1' s involvement in inflammatory regulation and its contribution contributes to the anti-inflammatory effects of butyrate.

### Regulation of RA inflammatory pathways and tissue damage by *Lactobacillus casei* combined with butyrate

**Figure 8.**
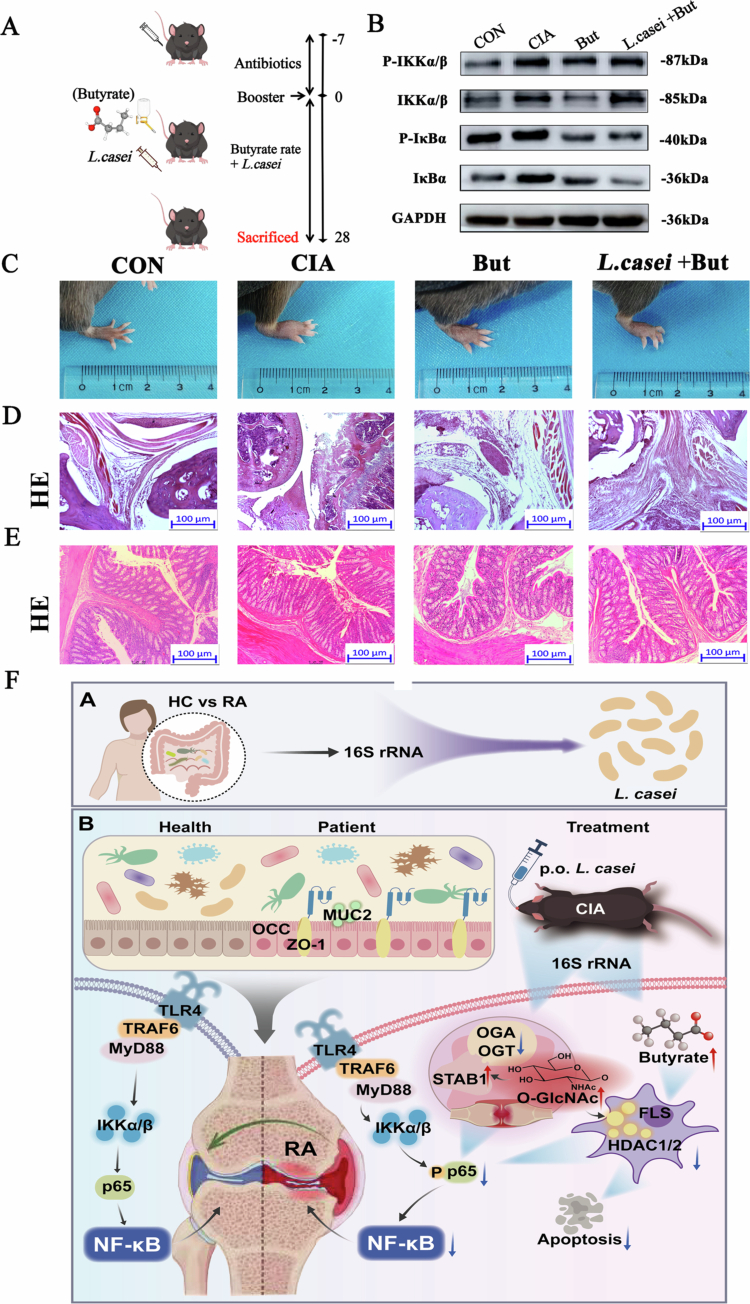
Regulation of RA inflammatory pathways and tissue damage by *L. casei* combined with butyrate. (A) Experimental protocol for evaluating the effects of *L. casei* + But co-administration on RA; (B) Western blot analysis of *p*-IKKα/β, IKKα/β, *p*-IκBα and IκBα levels after *L. casei* + But intervention; (C) Evaluation of paw swelling in CIA mice; (D) H&E staining analysis of ankle joints; (E) H&E staining analysis of colon; (F) Immunofluorescence staining of colonic barrier-associated proteins; (G) Schematic diagram of the protective effects of *L. casei* combined with butyrate in RA. Note: CON, normal control mice; CIA, collagen-induced arthritis mice; But, sodium butyrate. (*n* = 3, *p < 0.05; **p < 0.01).

Increased fecal butyrate levels were observed in the *L. casei*-H group via gas chromatography analysis (Figure S1D, Supporting Information). To further evaluate the synergistic effects of *L. casei* and butyrate, C57BL/6J mice were subsequently divided into four groups: CON, CIA, But and *L. casei* + But, and treated for 4 weeks (Figure 8A). Paw swelling was monitored throughout the intervention period (Figure 8C). Western blot analysis revealed markedly elevated levels of IKKα/β, IκBα, and their phosphorylated forms were markedly elevated in CIA mice. *L. casei* treatment significantly downregulated these proteins, with combined butyrate treatment further enhancing this inhibitory effect (Figure 8B, Figure S1E, Supporting Information). Consistently, inflammatory cytokines were reduced after treatment (Figure S2D-F, Supporting Information). H&E staining showed severe synovial hyperplasia, inflammatory infiltration, cartilage destruction, and intestinal villus damage in CIA mice. These pathological changes were partially ameliorated by *L. casei* and most effectively alleviated by *L. casei* + But treatment, restoring joint and intestinal structures (Figure 8D, E). Immunohistochemistry further confirmed reduced nuclear P-p65 expression after intervention (Figure S1F, Supporting Information). Collectively, these findings demonstrate that *L. casei* combined with butyrate synergistically suppresses NF-κB signaling and ameliorates joint and intestinal pathology in CIA mice (Figure 8F).

## Discussion

RA-associated gut microbial dysbiosis is characterized by alterations not only in microbial composition but also in systemic metabolic function and immunoregulatory capacity. This transforms the gut from a symbiotic, homeostatic environment into a potential promoter of inflammatory responses.^15^ Our study focused on the multi-level regulatory framework connecting gut microbiota, microbial metabolites, post-translational protein modifications, and inflammatory signaling pathways during RA progression. Consequently, microbial interventions are not merely about correcting microbial composition. Instead, they involve modulating metabolite production, regulating post-translational modification states, and remodeling signal transduction processes,^16^ thereby influencing host immune homeostasis and exerting sustained effects on inflammation initiation and progression.

Changes in gut microbial composition are closely coupled with metabolic reprogramming. In RA patients, gut microbiota alterations extend beyond compositional dysbiosis to include systemic remodeling of functional outputs, notably the overall suppression of short-chain fatty acid biosynthesis pathways.^17^ These function-oriented alterations, compared to differences in microbial abundance alone, more accurately reflect the microbiota' s actual involvement in disease initiation and progression.^18^ In our study, we found that *L. casei* significantly influenced O-GlcNAc modification levels and increased the production of UDP-GlcNAc, a key metabolite in the O-GlcNAc cycling pathway. This suggests that intracellular glycosylation status is highly sensitive to changes in the gut microecological environment. The hexosamine biosynthetic pathway, an important metabolic branch connecting nutrient sensing with post-translational modification, may thus serve as a critical bridge linking microbial metabolism to host immune regulation.^19^ Meanwhile, butyrate, a major microbial metabolite, not only maintains intestinal microenvironmental stability but also regulates intracellular substrate availability and associated modification processes.^20^ These findings are consistent with the recently proposed “microbiota-metabolism-immunity axis” model,^21^ further indicating that the gut microbiota' s role in RA has shifted from simple compositional association toward deeper regulation of host functional states.

We found that alterations in intestinal barrier function play a crucial, upstream role in integrating a multi-layered regulatory network. This network encompasses gut microbial dysbiosis, metabolic reprogramming, changes in post-translational modifications (such as O-GlcNAcylation and acetylation), and NF-κB inflammatory signaling. Restoration of tight junction proteins and mucus layer–associated molecules coincided with suppression of the TLR4/MyD88 signaling pathway, suggesting that gut-derived immune-inflammatory stimulation was effectively restricted, thereby reducing the persistent transmission of pro-inflammatory signals contributing to systemic inflammation in RA.^22^ Impairment of intestinal barrier integrity promotes the translocation of bacterial components and endotoxins into the circulation, a key mechanism underlying RA progression.^23^ Specifically, under disrupted barrier conditions, these products readily cross the epithelium into the lamina propria, activating pattern recognition receptors and inducing persistent downstream inflammatory signaling. This facilitates the transition from localized intestinal inflammation to systemic immune dysregulation and sustained systemic inflammatory responses.^24^ In the present study, we focused not only on alterations in tight junction proteins but also on the synchronized restoration of the mucus layer with the suppression of receptor-mediated signaling.^25^ This approach allowed us to examine regulation at the very source of immune activation, as evidenced by the reduced activity of the TLR4/MyD88 pathway, which suggests inflammatory responses were effectively restrained at the receptor signaling level.^26^

The NF-κB signaling pathway is regulated through multiple coordinated steps, rather than by a single isolated signaling node.^27^ These steps include receptor complex formation, IKK/IκBα phosphorylation status, and p65 nuclear translocation.^28^ Upstream, alterations in receptor complex formation indicate that inflammatory signaling is restricted during the initiation phase. Following signal transmission into the cytoplasm, suppression of the IKK/IκBα axis interrupts the NF-κB activation cascade.^29^ Additionally, changes in HDAC1 expression and activity may influence the transcriptional activity of p65 by regulating its acetylation status.^30^ In this study, we integrated these processes with the metabolic and post-translational modification network influenced by *L. casei*, suggesting that NF-κB regulation operates within a multidimensional system. Furthermore, O-GlcNAc modification introduces an additional regulatory dimension for inflammatory control. O-GlcNAc-modified proteins were primarily enriched in nuclear transcriptional regulatory domains and encompassed key signaling molecules, including NF-κB and HDAC-related regulatory components, indicating a central role in signal integration. Compared with phosphorylation and acetylation, O-GlcNAcylation exhibits a more dynamic and reversible nature,^31^ with modification sites predominantly occurring on serine or threonine residues, where they may compete or cooperate with phosphorylation events.^32^ Through modulation of NF-κB p65 activity, O-GlcNAc modification ultimately influences the transcription of inflammation-related genes.^33^

In this study, butyrate independently regulated HDAC- and NF-κB-related molecules in vitro, demonstrating anti-inflammatory effects even without a microbial background.^34^ The observed effects with the HDAC inhibitor TSA and the NF-κB inhibitor PDTC further linked butyrate-mediated regulation to classical inflammatory pathway inhibition. Synergistically, *Lacticaseibacillus casei* and butyrate enhanced the stability and persistence of anti-inflammatory effects by modulating NF-κB signal transduction, HDAC-associated epigenetic regulation, and the transcriptional processes of inflammation-related genes.^35^ Previous studies indicate that SCFAs may exert biological effects via both receptor-dependent and receptor-independent pathways. These pathways regulate immune cell activation, cytokine release, and chemotactic responses, thereby maintaining intestinal immune homeostasis. SCFAs also interact with classical inflammatory signaling pathways, such as NF-κB, influencing both the intensity and duration of inflammatory responses.^36^ In addition, these receptor systems may be closely coupled with host metabolic status, translating microbiota-derived signals into immunoregulatory signals and contributing to the systemic fine-tuning of inflammatory processes.^37^ Thus, at the molecular level, butyrate and its associated metabolic signaling pathways may coordinately regulate inflammation through both receptor-mediated and non-receptor mechanisms. However, the relative contributions of these pathways are not fully understood, and the dynamic interplay among O-GlcNAc modification, HDAC regulation, and NF-κB signaling requires further detailed investigation.

This study has several limitations that warrant further investigation. One important issue is the need to combine GPR receptor blockade strategies with gut microbiota butyrate-depletion interventions to more precisely define butyrate' s contribution to the anti-inflammatory effects mediated by *L. casei.* Additionally, the dynamic interactions between O-GlcNAcylation and other post-translational modifications are still insufficiently understood and require further validation using clinical samples. These future directions align with the current trend toward integrated “basic-to-clinical translational research” and may facilitate the translation of microbiota-based interventions into precision therapeutic strategies.

## Methods

### Materials and reagents

Low- and high-speed centrifuges (KDC-1044, KDC-160HR), a vortex mixer (Vortex-5), a small animal anesthesia machine (H1556801-039), a foot volume meter (PV-200), mini electrophoresis and transfer tanks (WIX-MiniPro2), and a chemiluminescence imager(LAS500) were utilized in the study, along with PDTC (5108-96-3) and TSA (58880-19-6, MedChemExpress) were also employed. Chromatographic separation was achieved using an ACQUITY BEH C18 column (100 × 2.1 mm, 1.7 µm). LC-MS-grade methanol and acetonitrile were obtained from Thermo Fisher Scientific, while bovine type II collagen (220195) and Freund's complete adjuvant (SLCL9648) were supplied by Sigma-Aldrich.

### Collection of clinical data

The clinical study was approved by the Institutional Ethics Committee, and written informed consent was obtained from all participants. Eligible RA patients were aged ≥18 years, had disease duration >6 months, met established diagnostic criteria, and fulfilled active disease criteria based on DAS28 or inflammatory markers. Exclusion criteria included recent use of antibiotics, probiotics, or gastrointestinal motility drugs were excluded. At enrollment, baseline demographic and clinical data were recorded, including age, sex, BMI, smoking history, dietary habits and medication history. Laboratory parameters measured included C-reactive protein (CRP), IL-6, TNF-*α*, IL-8 and rheumatoid factor (RF). The Disease Activity Score in 28 joints (DAS28) was also determined. Disease activity was classified as remission (DAS28 < 2.6), low (2.6 ≤ DAS28 ≤ 3.2), moderate (3.2 < DAS28 ≤ 5.1), or high (DAS28 > 5.1).

### Animal grouping and modeling

All animal experiments were approved by the Institutional Animal Care and Use Committee of Shanxi University of Chinese Medicine and adhered to institutional guidelines. Male C57BL/6J mice (SPF grade, 6 weeks old, 18–20 g) were obtained from Vitonlihua Experimental Animal Technology Co., Ltd (Beijing, China; License No. SCXK [Jing] 2021–0006; Certificate No. 110011241103887151). The mice were housed under SPF conditions, maintained at 20−26 °C, 40–70% humidity, 12 h light/dark cycle, allowing them free access to food and water.

Following a 7-day acclimatization period, the mice were randomly assigned to four groups: control, CIA model, high-dose *L. casei* (1 × 10^9^ CFU/mL, 0.1 mL), and low-dose *L. casei* (1 × 10^8^ CFU/mL, 0.1 mL). CIA was induced in all groups, excluding controls, by intradermal injection of an emulsion of bovine type II collagen and complete Freund's adjuvant (CFA) (0.1 mL per mouse) into the right hind paw, back, and tail root under 1.5% isoflurane anesthesia. Control mice received an equivalent volume of saline (0.1 mL) at the same sites. A booster injection of the collagen-CFA emulsion or saline (0.1 mL) was administered on day 7, which was subsequently designated as day 1 for the study timeline. Starting from day 1, mice received daily oral gavage of L. casei or distilled water for 4 weeks. Body weight and paw thickness were recorded weekly thereafter.

### Gathering of samples

At the conclusion of the experiment, fecal samples were collected immediately prior to euthanasia. Subsequently, blood and various tissues—including the spleen, lung, thymus, knee joint, synovium, colon, and paw—were harvested. Portions of the paw, knee joint, synovium, and colon were fixed in 4% paraformaldehyde for histological analysis. The remaining tissues were snap-frozen in liquid nitrogen and stored at −80 °C for subsequent analyzes.

### Monitoring joint swelling and body weight in mice

Ankle circumference and body weight were measured weekly using a vernier caliper. Joint swelling was quantified as a percentage increase relative to baseline measurements, calculated using the following formula: swelling degree = [(ankle circumference at measurement - baseline value)/baseline value] × 100%.

### Small animal in vivo imaging protocol

Mice were anesthetized with isoflurane and depilated prior to imaging. Bioluminescent substrates (hydrogen peroxide probe) were administered subcutaneously and allowed to distribute for a period ranging from 15 min to 48 h. Subsequently, the animals were placed on a temperature-controlled imaging platform (e.g., IVIS Spectrum, PerkinElmer) for image acquisition. Images were acquired using specified parameters, including exposure time, aperture, and excitation/emission wavelengths. Fluorescence or luminescence intensity in designated regions of interest was then quantified using analysis software (e.g., Living Image). To ensure consistency, control groups were included, and the duration of anesthesia, probe dosage, and imaging conditions were standardized. All procedures were conducted in a light-protected environment, adhering to animal ethics guidelines.

### MicroCT

Following a 28-day treatment, the mice were euthanized, and their left ankle joints and paws were collected and fixed in 4% formalin for one week. The specimens were then scanned using a SkyScan 1174 microCT (Bruker, Belgium). After scanning, data were reconstructed in 3D using *N*-Recon software and analyzed with CT-An to determine bone volume (BV) and bone surface (BS). The BS/BV ratio was calculated to evaluate changes in bone surface density and microstructure.

### Cell growth

Fibroblast-like synoviocytes (FLS, CTCC-S004-MIC) were obtained from Zhejiang Meisen Cell Technology Co., Ltd. Cells were cultured under standard conditions in DMEM (Gibco, 8123617) supplemented with 10% fetal bovine serum (FBS, Gibco, 10099141 C) and antibiotics (100 U/mL penicillin and 0.1 mg/mL streptomycin).

### Assay for wound healing

FLS were cultured in DMEM enriched with 10% FBS and 1% penicillin/streptomycin at 37 °C in an atmosphere of 5% CO₂. Once the cells reached 80-90% confluence, they were stimulated with IL-1β and TNF-*α* at a concentration of 100 nM each for 24 h. Log-phase cells, at a density of 1.5 × 10^6^ per well, were then seeded into 6-well plates. Wounds were created using a 200 μL pipette tip. Following a wash with PBS, the cells were incubated in serum-free medium containing various concentrations of heat-inactivated *L. casei* (0, 5, 10, and 20 mg/mL) for 12 h. Wound closure was monitored at 0 and 12 h using inverted microscopy. All experiments were conducted in triplicate.

### Flow cytometry

FLS were cultivated to the logarithmic phase, subsequently harvested, and resuspended in PBS (at a concentration of 1 × 10^6^ cells/mL). The cells were then incubated in 1 × binding buffer and stained with FITC Annexin V, followed by apoptosis analysis using flow cytometry.

### Immunofluorescence (IF) staining

After 48 h of CCD treatment, FLS were fixed with 4% paraformaldehyde for 20 min at room temperature. Cells were then permeabilized with 0.3% Triton X-100 and blocked with 10% goat serum. Cells were incubated overnight at 4 °C with anti-NF-κBp65 antibody (1:4000, Proteintech, 10745-1-AP), followed by a 1 h incubation with Cy3-conjugated secondary antibody (1:1000). Nuclei were counterstained with DAPI, and images were captured and merged using ImageJ.

### Masson, AB-PAS staining

Joint tissues were fixed in 4% paraformaldehyde, paraffin-embedded, cleared with xylene, and dehydrated in graded ethanol. Sections (4 μm) were then cut with a microtome and stained using Masson trichrome (Solarbio, 20230323) and Alcian Blue-PAS kits (Solarbio, 20230324) following the manufacturers' instructions.

### Immunohistochemistry

For immunohistochemical staining, mouse intestinal tissues were processed using a commercial IHC kit (Wuhan Boster Biological Technology., LTD, 16l23e28k1522). Tissues were fixed in 4% paraformaldehyde, paraffin-embedded, sectioned, deparaffinized, and rehydrated. Endogenous peroxidase activity was blocked, followed by heat-mediated antigen retrieval. The sections were then blocked with 5% BSA and incubated overnight at 4 °C with the primary antibody. Subsequently, they were incubated with an HRP-conjugated secondary antibody for 1 h. The signal was developed using DAB and counterstained with hematoxylin. Images were acquired via microscopy and quantified with ImageJ.

### Staining with ghost pen cyclic peptides

FLS were fixed with paraformaldehyde for 10 min, followed by a 5-min permeabilization with 0.1% Triton X-100 to facilitate the entry of the Ghost Pen Peptide into the cells and its binding to F-actin. The samples were then incubated in the dark with a working solution of the fluorescent probe ghost pen peptide (C2203S, Beyotime) at a concentration of 50–200 nM for 1 h. Nuclei were counterstained with DAPI for 5 min, and fluorescence images were captured with a fluorescence microscope.

### Transmission electron microscopy

Following fixation with glutaraldehyde and osmium tetroxide, the samples were dehydrated in graded ethanol and embedded in epoxy resin. After polymerization, ultrathin sections (60-80 nm) were prepared, stained with uranyl acetate and lead citrate, and examined with a transmission electron microscope (H-7650, 80 kV) equipped with a CCD camera (Gatan 830).

### Western blot

Protein concentrations were measured using a BCA assay(AR0146, Bosterbio) after lysis in RIPA buffer (AR0102, Bosterbio) with protease (PR20032) and phosphatase inhibitors (PR20015, Proteintech) were utilized in the experimental protocol. Equal quantities of protein were separated via SDS-PAGE (AR0138, Bosterbio) and subsequently transferred to PVDF membranes (Millipore, USA). The membranes were blocked with 5% skim milk in TBST, followed by overnight incubation at 4 °C with primary antibodies against HDAC1 (1:10000, 10197-1-AP, Proteintech), HDAC2 (1:10000, 12922-3-AP, Proteintech), Occludin (1:8000, Proteintech, 13409-1-AP), IKKα/β (1:1000, Boster, BM4499), *p*-IKKα/β (1:500, Abmart, PC3237S), IκBα (1:30000, Abmart, PC1287S), *p*-IκBα (1:500, Abmart, TP70389S), TLR4 (1:1000, A5258, ABclonal), MyD88 (1:100067969-1-Ig, Proteintech), TRAF6 (1:2000, A16991, ABclonal), NF-κB p65 (1:4000, Proteintech, 10745-1-AP), *p*-NF-κB p65 (1:1000, Proteintech, 10268-1-AP), ZO-1 (1:5000, Proteintech, 21773-1-AP), STAB-1 (1:4000, Abmart, PK87221S), and *β*-actin (1:5000, Bioworld Technology, USA, AP 0060). Subsequently, membranes were incubated with HRP-conjugated goat anti-rabbit IgG (1:5000, Proteintech) for 2 h at room temperature. Bands were visualized using Meilunbio ® FG Super Sensitive ECL reagent (MA0186) and quantified by densitometric analysis with ImageJ.

### ELIZA

Serum levels of IL-1β, IL-6, and TNF-*α* were quantified using commercial mouse ELIZA kits. Absorbance was measured at 450 nm with an ELIZA microplate reader, and cytokine levels were calculated using standard curves.

### siRNA transfection

RA-FLS cells were transfected with STAB1-specific siRNA using Lipofectamine 3000 (Invitrogen, USA) according to the manufacturer’s instructions. The STAB1 siRNA duplexes were synthesized by GenePharma (Shanghai, China). The sequences were as follows:Sense: 5′-CAGUGGUUUAAGAACUCGA[dT][dT]-3′ Antisense: 5′-UCGAGUUCUUAAACCACUG[dT][dT]-3′. Briefly, cells were seeded in 6-well plates and transfected after reaching 60–70% confluence. Following 24 h of transfection, cells were treated with sodium butyrate for the indicated time period and subsequently collected for western blot. Knockdown efficiency was evaluated by western blotting.

### Protein isolation and peptide breakdown

Proteins were isolated using UA lysis buffer and quantified by Bradford assay. For peptide breakdown, 20 µg aliquots of the proteins were first heated with 5 × loading buffer and resolved by SDS-PAGE. The resulting samples were then reduced with DTT, alkylated with IAA, and diluted to 2 M. Following these steps, they were digested overnight with trypsin, acidified to pH ≤ 3 with TFA to a pH of ≤3, purified using C18 SPE, and finally lyophilized.

### Enrichment of O-GlcNAcylated peptides

Prior to enrichment, the quality of protein extraction and digestion was pre-assessed via 12.5% SDS-PAGE to ensure efficient enrichment. For enrichment, Lyophilized peptides were reconstituted in 1.4 mL of cold IAP buffer and incubated overnight at 4 °C with an anti-O-GlcNAc monoclonal antibody (CTD110.6) conjugated to Protein A/G Plus-Agarose beads (sc-2003, Santa Cruz Biotechnology, Dallas, TX, USA). After incubation, the beads were washed three times with IAP buffer and twice with deionized water to remove non-specific binders. O-GlcNAcylated peptides were then eluted twice with 0.15% TFA, desalted using C18 StageTips (ZipTip), and lyophilized for subsequent 4D-Label-free LC-MS/MS analysis on a timsTOF Pro mass spectrometer (Bruker Daltonics). The quality of protein extraction and digestion was pre-assessed via 12. 5% SDS-PAGE to ensure enrichment efficiency.

### GC data acquisition

GC analysis was performed using an HP-5MS capillary column (30 m × 0.250 mm, 0.25 µm, Agilent J&W GC Columns) with helium (≥99.999%) as the carrier gas at 1 mL/min. A 1 μL sample was automatically injected in split mode (10:1), with the injection port set at 290 °C. The oven temperature program was as follows: an initial hold at 40 °C for 2 min, followed by an increase to 150 °C at 15 °C/min (1 min), and finally a rapid ascent to 300 °C at 30 °C/min, maintained for 5 min.

### 16S rRNA gene sequencing and bioinformatics analysis

Total genomic DNA was extracted from fecal samples using the MagBeads FastDNA Kit for Soil. The V3-V4 regions of the 16S rRNA gene were PCR-amplified using primers 338F and 806 R, and subsequently sequenced on the Illumina NovaSeq 6000 platform. Microbiome bioinformatics were performed using QIIME2 (version 2022.11). Briefly, raw sequences underwent quality filtering, denoising, and merging via the DADA2 plugin to generate Amplicon Sequence Variants (ASVs). ASVs were taxonomically assigned using a naïve Bayes classifier against the Greengenes Database (Release 13.8). Alpha and beta diversity analyzes were then conducted based on the ASV table, and differential taxa were identified using MaAsLin2 (FDR < 0.05).

### Cluster evaluation of altered peptide fragments

After normalizing target protein data to (−1, 1), hierarchical clustering heatmaps were then generated using the ComplexHeatmap R package (R 3.4), employing Euclidean distance and average linkage for both samples and proteins.

### Analysis of subcellular localization

Protein subcellular localization was predicted using CELLO (http://cello.life.nctu.edu.tw/). This tool employs multi-class support vector machines (SVMs) that are trained on proteins with known localization to infer the locations of target proteins.

### Domain architecture characterization

Domain architecture was analyzed using the InterProScan suite, an integrated platform that combines signature-recognition engines from the InterPro resource. This suite utilizes the Pfam repository, an archive of protein families defined by curated alignments and hidden Markov model profiles, to translate primary sequences into functional units and retrieve Pfam-derived domain footprints for the query proteins.

### Enrichment analysis

Enrichment of GO terms, KEGG pathways, and Pfam domains was quantified using a two-tailed Fisher's Exact Test. The test compared the prevalence of these annotations in the focal protein cohort to the genomic background. Only over-represented annotations that survived correction for multiple testing (FDR < 0.05) were considered significant.

### Statistical analysis

All experiments were performed independently at least three times. Data are presented as mean ± SEM. Statistical analyzes were conducted using GraphPad Prism version 8.0 and IBM SPSS Statistics version 29.0. Pairwise comparisons were assessed using Student’s t-test. Differences among three or more groups were analyzed using one-way analysis of variance (ANOVA) followed by Tukey’ s multiple comparison test. A *p*-value of < 0.05 was considered statistically significant (*p < 0.05, **p < 0.01, ***p < 0.001).

## Supplementary Material

Supporting Information2.docx Supporting Information2.docx

Author Agreement Statement.pdf Author Agreement Statement.pdf

Ethics Approval Letter.pdf Ethics Approval Letter.pdf

Informed Consent Statement.pdf Informed Consent Statement.pdf

Supporting Information1.docx Supporting Information1.docx

## Data Availability

The datasets generated and analyzed during the current study are available from the corresponding author upon reasonable request. The 16S rRNA gene sequencing data generated during the current study will be deposited in the NCBI Sequence Read Archive (SRA) and made publicly available upon acceptance of the manuscript. Synovial tissue transcriptomic data were obtained from NCBI’s Gene Expression Omnibus (GEO) database under the accession number GSE77298.
