# Supplementary material for: Probiotic Lactobacillus casei improves immune microenvironment in rheumatoid arthritis via gut microbiota-butyrate-HDAC/NF-κB signaling
Source: Gut Microbes. 2026 Jul 21;18(1):2698969. doi: 10.1080/19490976.2026.2698969 (PMC13393233; doi:10.1080/19490976.2026.2698969)
Supplement: Supporting Information2.docx [file KGMI_A_2698969_SM1740.docx]

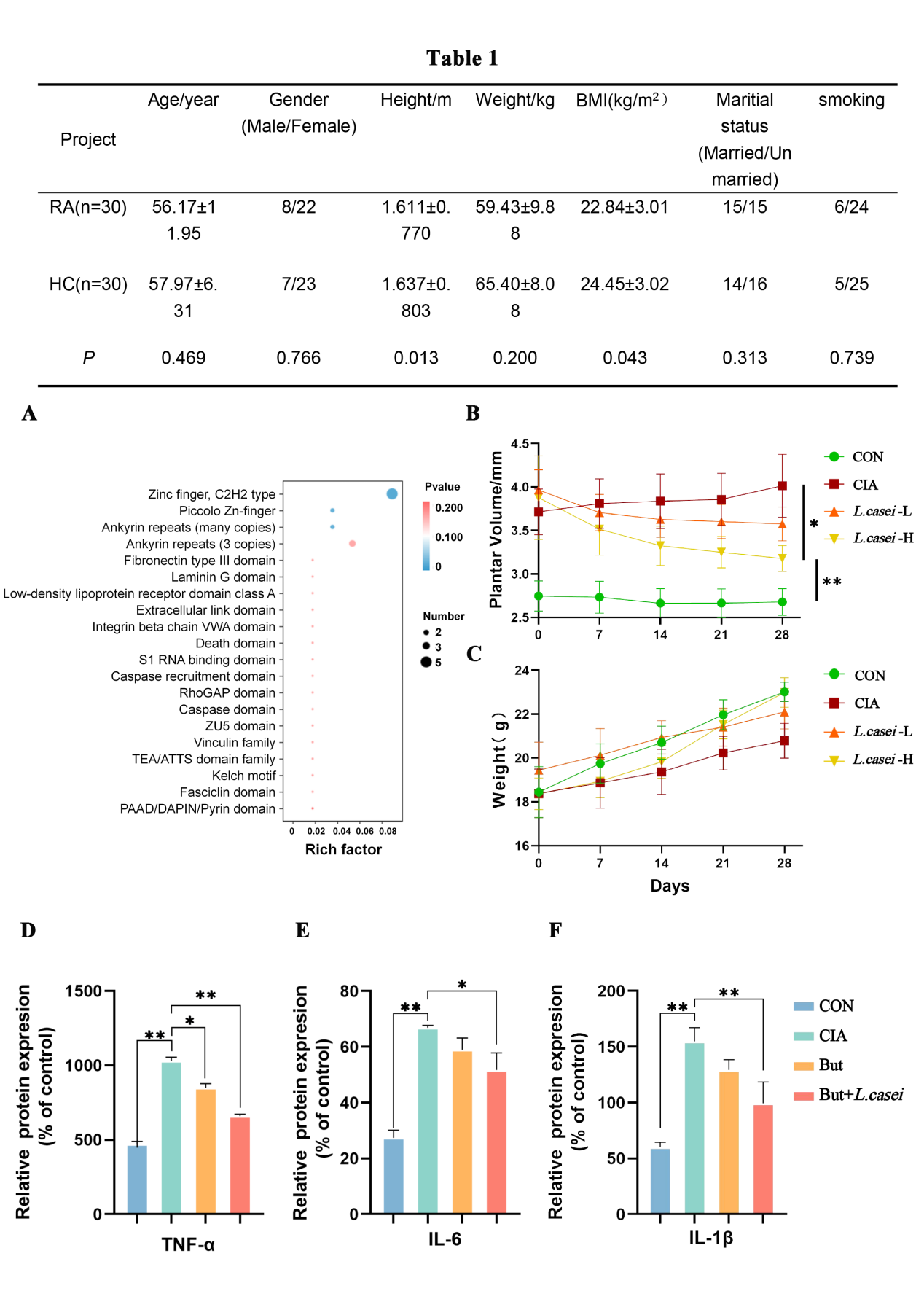


**Figure S2.**（Table 1）Baseline Data Table for Clinical Patients and Healthy Subjects; Data are presented as mean ± SD or n. Statistical comparisons were performed using Student’s t-test or chi-square test as appropriate. (A) KEGG enrichment analysis of glycosylation-differentiated peptides; (B) The effect of LPC on joint swelling in CIA mice; (C) The effect of LPC on body weight in CIA mice; (D) Detection of TNF-α expression in mouse blood by ELISA; (E) Detection of IL-6 expression in mouse blood by ELISA; (F) Detection of IL-1β expression in mouse blood by ELISA. **p* <0.05; ***p* <0.01.
