# Supplementary material for: Probiotic Lactobacillus casei improves immune microenvironment in rheumatoid arthritis via gut microbiota-butyrate-HDAC/NF-κB signaling
Source: Gut Microbes. 2026 Jul 21;18(1):2698969. doi: 10.1080/19490976.2026.2698969 (PMC13393233; doi:10.1080/19490976.2026.2698969)
Supplement: Author Agreement Statement.pdf [file KGMI_A_2698969_SM1741.pdf]

Author Agreement Statement：

We, the undersigned, hereby solemnly affirm that this manuscript constitutes original scholarly work, has not heretofore been published in any medium or format and is not presently under consideration for publication by any other journal or publishing entity.

We attest that all authors enumerated herein have critically reviewed and unanimously endorsed the final version of this manuscript. We further certify that no individuals satisfying established authorship criteria have been omitted from this submission and that the sequence of authors has been deliberated upon and expressly sanctioned by all contributors.

We understand that the Corresponding Author is the sole contact for the Editorial process. He/she is responsible for communicating with the other authors about progress, submissions of revisions and final approval of proofs Signed by all authors as follows:

Yanmiao Ma, Mingyang Li, Liyuan Jian,
Jiehao Peng, Ya Wen, Yuanhui Hao,
Jiajia Shen, Yuli Song, Bowzhen Li,
Chengwu Zheng, Xinyu Li, Guganglin Li,
Yonghui Wang, Tao Peng, Ran Zhou
