# Supplementary material for: Probiotic Lactobacillus casei improves immune microenvironment in rheumatoid arthritis via gut microbiota-butyrate-HDAC/NF-κB signaling
Source: Gut Microbes. 2026 Jul 21;18(1):2698969. doi: 10.1080/19490976.2026.2698969 (PMC13393233; doi:10.1080/19490976.2026.2698969)
Supplement: Ethics Approval Letter.pdf [file KGMI_A_2698969_SM1739.pdf]

长治医学院附属和平医院医学伦理委员会

伦理审查批件

批件号	（2025）049号				
项目名称	基于宏基因组学探讨风湿宁干预肠道菌群治疗类风湿关节炎的临床研究				
临床研究类别	生物医学研究				
研究单位	长治医学院附属和平医院				
主要研究者	宋亚丽				
申办单位	无				
审查类别	初始审查	审查日期	2025年6月6日	审查方式	快速审查
审查委员	张鹏飞 王治平				
审查文件	1、初始伦理审查申请表<br>2、临床研究方案<br>3、知情同意书<br>4、病例报告表<br>5、主要研究者及研究团队利益冲突声明<br>6、研究者专业履历及GCP证书				

表决结果汇总				
投票人数	2 人		回避人数	0 人
投票结果	同意 2票	不同意 0票	作必要修改后同意 0票	作必要修改后重审 0票
结论	☑ 同意	☐ 不同意	☐ 作必要修改后同意	☐ 作必要修改后重审

审查意见

根据卫生部《涉及人的生物医学研究伦理审查办法（2016）》、中国医院协会《涉及人的临床研究伦理审查委员会建设指南（2019版）》、WMA《赫尔辛基宣言》和CIOMS《人体生物医学研究国际道德指南》的伦理原则，经本伦理委员会审查，同意按所批准的临床研究方案、知情同意书开展本项研究。

请遵循GCP原则、遵循伦理委员会批准的方案开展临床研究，保护受试者的健康与权力。

研究过程中若变更主要研究者，对临床研究方案、知情同意书等的任何修改，请申请人提交修正方案审查申请。

发生严重不良事件，请申请人及时提交严重不良事件报告。

请按照伦理委员会规定的跟踪审查频率，申请人在截止日期前1个月提交研究进展报告；当出现任何可能显著影响试验进行、或增加受试者危险的情况时，请申请人及时向伦理委员会提交书面报告。

研究过程中发生不依从/违背研究方案，或可能对受试者的权益/健康以及研究的科学性造成不良影响等违背GCP原则的情况，请申办者/监察员/研究者提交不依从/违背研究方案报告。

申请人暂停或提前终止临床研究，请及时提交暂停/终止研究报告。

完成临床研究，请申请人提交研究完成报告。

本试验（基于宏基因组学探讨风湿宁干预肠道菌群治疗类风湿关节炎的临床研究）应在批准后1年内实施，逾期未实施本批件自行废止。

跟踪审查频率	自本试验批准之日起 ☑ 1年；☐ 6个月； ☐其他________
联系地址	地址：山西省长治市延安南路110号<br>邮编：046000<br>电话：0355-3128042<br>邮箱：czhpk.jk@163.com
联系人	武平
主任委员签字	[illegible]
伦理委员会	（盖章）
日期	2015年6月6日
