# Supplementary material for: Probiotic Lactobacillus casei improves immune microenvironment in rheumatoid arthritis via gut microbiota-butyrate-HDAC/NF-κB signaling
Source: Gut Microbes. 2026 Jul 21;18(1):2698969. doi: 10.1080/19490976.2026.2698969 (PMC13393233; doi:10.1080/19490976.2026.2698969)
Supplement: Informed Consent Statement.pdf [file KGMI_A_2698969_SM1743.pdf]

知　情　同　意　书

尊敬的病友：

您现在所患疾病是＿类风湿关节炎＿我们邀请您参加一项临床研究。参加这项研究完全是您自主的选择。本知情同意书将提供给您一些信息，请您仔细阅读，并慎重做出是否参加本想研究的决定。如有任何关于本项研究的疑问，您可以请您的医生或研究人员给予解释。您可以和家人及朋友讨论，以帮助您决定是否自愿参加此项临床研究。您有权拒绝参加本研究，也可随时退出研究，且不会受到处罚，也不会失去您应有的权利。

如果您同意参加，我们将需要您签署本知情同意书并注明日期。您将获得一份已签字并注明日期的副本，供您保存。

您参加本次研究是自愿的，本项研究已通过本院医学伦理委员会审查。

【研究名称】

【研究单位】长治医学院附属和平医院

【主要研究者】宋亚丽

【研究资助者】长治医学院附属和平医院

【为什么要进行该项研究？】

基于宏基因组学分析微生物与宿主之间的协作关系，明确风湿宁是否通过调节肠道菌群及其衍生物丰度结构治疗类风湿关节炎。并对风湿宁治疗类风湿关节炎患者相关临床症状、生活质量等情况的影响进行临床疗效及安全性评价，探索肠道菌群与临床指标之间的相关性，为风湿宁在临床中的进一步应用提供理论指导。

患者可以获取关于自身疾病的详细分析，能获得更全面的健康状况评估，为早期诊断和个性化治疗提供参考。研究期间的粪便宏基因组检测费用及风湿宁费用由项目承担，患者无需支付额外费用。

【本研究如何进行？】

收集本院风湿免疫科门诊确诊的类风湿关节炎患者经风湿宁治疗前后的粪便与血液，进行宏基因组测序与临床指标检测，肠道菌群与临床指标之间的相

关性，阐明风湿宁治疗 **RA** 的肠道微生态机制。

【参加研究的条件】

1. 入选标准为：①符合《中医康复临床实践指南·类风湿关节炎》中对于类风湿关节炎的诊断标准；②年龄≥**18** 岁；③患者病程＞**6** 个月；④至少一月内未服用益生菌、抗生素、胃肠动力药物；⑤筛选时符合中医风寒湿痹证辨证标准，符合活动性类风湿关节炎（**DAS28-ESR>2.6** 或 **DAS28-CRP>2.6**）诊断，或 **C** 反应蛋白或红细胞沉降率大于正常值上限；⑥无相关药物过敏史；⑦患者临床病例资料齐全，家属均知晓本研究，签署知情同意书。

2. 排除标准为：①患有其他免疫系统疾病；②存在内分泌系统疾病；③心肝肾功能异常；④对中药过敏或不能耐受中药，不能坚持服用中药者；⑤存在精神障碍性疾病。

3. 中途退出标准为：患者因任何原因不再想继续参与试验，可随时退出。

【我参加本研究的时间将有多长？】

完成相关检查，约 2 周时间。

【我有哪些责任与义务】

本研究将会对您的病情和诊断做一详尽的评估，如符合我们的纳入标准，我们将在征得您知情同意的基础上采集血粪便、血液标本，于试验开始后服用中药复方风湿宁 2 周，并在试验前后填写相关量表，其他无对您生活存在影响的问题。如果您有关于研究中其他任何疑问可以向研究医生咨询。

【参加本研究可能有哪些风险？】

本研究对受试者粪便基因测序结果以及临床指标进行分析，过程安全，受试者仅在血液标本采集过程中有疼痛、皮下血肿的可能。

【参与本研究可能获得什么益处？】

确诊患者可明确肠道菌群多样性及丰度变化情况、临床指标检测结果，达到早期诊断，早期治疗。

【我需要支付什么费用？】

无需支付正常诊疗之外的额外费用。

【研究相关伤害的医疗和赔偿】

如发生与本研究相关的损害，经国家法律法规规定的权威机构认定需要承担相应责任的，项目组将为您提供免费的治疗，按照国家法律法规进行赔偿。

【如果我不想参加本研究或者中途退出研究，会怎样？】

您可以选择不参加本项研究，或者在任何时候通知研究者要求退出研究，您的数据将不纳入研究结果，您的任何医疗待遇与权益不会因此而受到影响。

【我的个人信息会如何处理？】

如果您决定参加本项研究，您参加研究及在研究中的个人资料均属保密。您的样本将以研究编号数字而非您的姓名加以标识。可以识别您身份的信息将不会透露给研究小组以外的成员，除非获得您的许可。您的档案仅供研究人员查阅。为确保研究按照规定进行，必要时，政府管理部门或伦理审查委员会的成员按规定可以在研究单位查阅您的个人资料。这项研究结果发表时，将不会披露您个人的任何资料。

【我可以联系哪些人员，以便详细了解本研究？】

如果您在研究过程中，需要进一步了解有关研究资料信息，或您在任何时候觉得自己的任何症状给您造成问题，或如果您遭受研究相关损伤，请联系您的研究医生/研究人员__宋亚丽__，电话 15035590459 。

【我可以联系哪些人员，了解我作为研究受试者享有的权利？】

本知情同意书以及本研究已获得长治医学院附属和平医院医学伦理委员会（EC）的批准。EC 是一个有科研人士和非科研人士组成的团体，监督涉及人体受试者的研究。如果您对自己作为研究受试者所享有的权利存有任何疑问，请联系：长治医学院附属和平医院医学医学伦理委员会（0355-3128036）。

【同意声明】

我已阅读了本知情同意书。

我有机会提问而且所有问题均已得到解答。

我理解参加本项研究是自愿的。

我可以选择不参加本项研究，或者在任何时候通知研究者后退出而不会遭到歧视或报复，我的任何医疗待遇与权益不会因此而受到影响。

如果我需要其它治疗，或者我没有遵守研究计划，或者发生了与研究相关的损伤或者有任何其它原因，研究医师可以终止我继续参与本项研究。

我同意参加本项临床研究并收到一份签过字的“知情同意书”副本。

患者（受试者）姓名（正楷）：______________　联系电话：______________

患者（受试者）签名：________________　　　日期：____年___月___日

患者（受试者）法定代理人姓名（正楷）：_______________

患者（受试者）法定代理人签名：______________　日期：____年___月___日

与患者（受试者）的关系：_________________

患者（受试者）法定代理人联系电话：____________________

研究者姓名（正楷）：________________

研究者签名：_________________　　　日期：____年____月___日

（注：如果受试者不识字时尚需见证人签名，如果受试者无行为能力时则需代理人签名）
