# Supplementary material for: Probiotic Lactobacillus casei improves immune microenvironment in rheumatoid arthritis via gut microbiota-butyrate-HDAC/NF-κB signaling
Source: Gut Microbes. 2026 Jul 21;18(1):2698969. doi: 10.1080/19490976.2026.2698969 (PMC13393233; doi:10.1080/19490976.2026.2698969)
Supplement: Supporting Information1.docx [file KGMI_A_2698969_SM1742.docx]

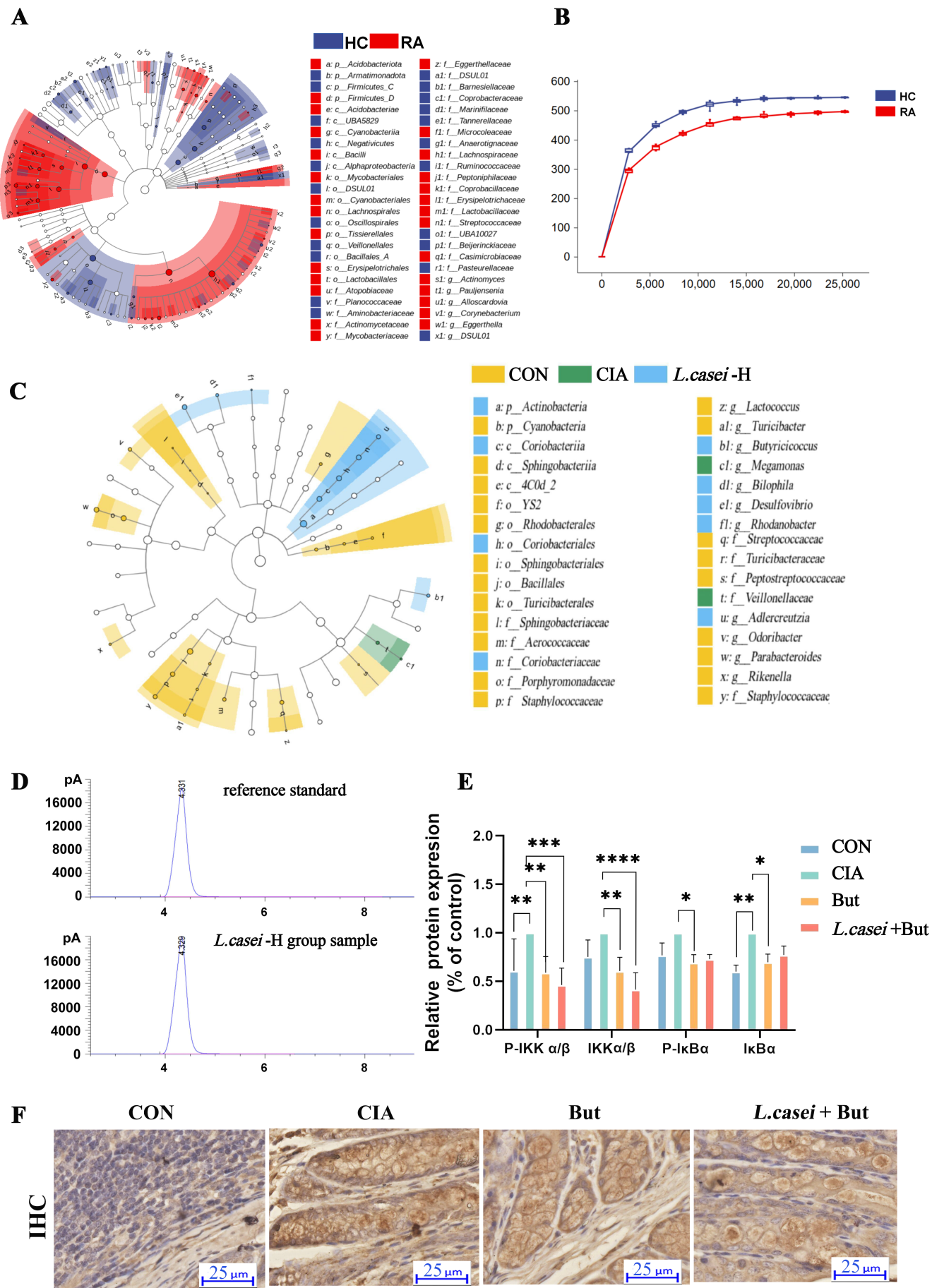


**Figure S1.** (A) Evolutionary branch diagram of microbial abundance changes between groups; (B) Rarefaction curve of gut microbiota in clinical patients and patients; (C) Evolutionary branch diagram of microbial abundance changes among groups; (D) Gas chromatography (GC) analysis of fecal samples from LPC-H group mice; (E) Western blot analysis of IKKα, IκBα, p-IKKα/β, and p-IκBα expression levels after LPC+But intervention; (F) p-p65 protein immunohistochemical detection; **p* <0.05; ***p* <0.01; ****p* <0.001; *****p* <0.0001.
